# Effects of acute hypoxia exposure with different durations on activation of Nrf2-ARE pathway in mouse skeletal muscle

**DOI:** 10.1371/journal.pone.0208474

**Published:** 2018-12-04

**Authors:** Weixiu Ji, Linjia Wang, Shiyi He, Lu Yan, Tieying Li, Jianxiong Wang, Ah-Ng Tony Kong, Siwang Yu, Ying Zhang

**Affiliations:** 1 School of Sport Science, Beijing Sport University, Beijing, China; 2 Tianjin University of Sport, Tianjin, China; 3 Faculty of Health, Engineering, and Sciences, University of Southern Queensland, Toowoomba, Queensland, Australia; 4 Ernest Mario School of Pharmacy, Rutgers, the State University of New Jersey, Camden, New Jersey, United States of America; 5 Department of Chemical Biology, School of Pharmaceutical Sciences, Peking University, Beijing, China; University of PECS Medical School, HUNGARY

## Abstract

**Background:**

Hypoxia training enhances the endurance capacity of athletes. This response may in part be attributed to the hypoxia-induced increase in antioxidant capacity in skeletal muscles. Nuclear factor erythroid 2-related factor 2 (Nrf2), a key transcription factor which regulates the expression of genes via binding to the antioxidant-response element (ARE) of these genes, plays a crucial role in stimulating the body’s defense system and potentially responds to hypoxia. Meanwhile, hypoxia-inducible factor-1α (HIF-1α) is an important player in protecting cells from hypoxic stress. The purpose of this study was to investigate the effects of acute hypoxia exposure with different durations on the activation of Nrf2-ARE pathway and a possible regulatory role of HIF-1α in these responses.

**Methods:**

C57BL/6J mice were allocated into the non-hypoxia 0-hour, 6-hour, 24-hour, and 48-hour hypoxic exposure (11.2% oxygen) groups. The quadriceps femoris was collected immediately after hypoxia. Further, to investigate the possible role of HIF-1α, C2C12 myoblasts with HIF-1α knockdown by small interfering RNA (siRNA) and the inducible HIF-1α transgenic mice were employed.

**Results:**

The results showed that 48-hour hypoxia exposure up-regulated protein expression of Nrf2, Nrf2/ARE binding activity and the transcription of antioxidative genes containing ARE (*Sod1* and others) in mouse skeletal muscle. Moreover, HIF-1α siRNA group of C2C12 myoblasts showed a remarkable inhibition of Nrf2 protein expression and nuclear accumulation in hypoxia exposure for 72 hours compared with that in siRNA-Control group of the cells. In addition, HIF-1α transgenic mice gave higher Nrf2 protein expression, Nrf2/ARE binding activity and expressions of Nrf2-mediated antioxidative genes in their skeletal muscle, compared with those in the wild-type mice.

**Conclusions:**

The findings suggested that the acute hypoxia exposure could trigger the activation of Nrf2-ARE pathway, with longer duration associated with higher responses, and HIF-1α expression might be involved in promoting the Nrf2-mediated antioxidant responses in skeletal muscle.

## Introduction

It has been demonstrated that exercise training under stimulated hypoxia conditions or at high altitude can increase endurance capacity in skeletal muscles of athletes [1,2]. Several studies have shown that endurance exercise training under hypoxia conditions significantly reduced the basal oxidants production, increased the expression of antioxidant proteins, and upregulated glutathione (GSH) system in skeletal muscle of humans or rats, compared to the outcomes from exercise training in normoxia conditions [3,4]. These results imply that the enhanced endurance capability may, at least in part, attribute to the hypoxia-induced increase in antioxidant capacity in skeletal muscles. Considering the potential effects of hypoxia on promoting anti-oxidation of skeletal muscle and improving athlete performance, it is important to investigate the molecular mechanism by which hypoxia affects antioxidant reaction in skeletal muscle.

Nuclear factor erythroid 2-related factor 2 (Nrf2) is one of the key transcription factors that stimulates body’s defense system and possibly responds to hypoxia [5,6]. The body defense system equips with the capacity to increase the expression levels of cytoprotective enzyme genes through Nrf2, which can bind to antioxidant response elements (ARE) located in the regulatory regions of many defense enzyme genes [5]. Downstream analysis has shown that Nrf2 target genes encode enzymes involved in a wide range of cellular defense processes, therefore, Nrf2 may enhance the overall capacity of cells to handle harmful substances and situations [7–9]. In addition, Nrf2-ARE signaling pathway may respond to hypoxic stimuli. It has been reported that hypoxic preconditioning had a neuroprotective effect on rat traumatic brain injury by increased expressions of Nrf2 and heme oxygenase1 (*Hmox1*) [10]. Similar protective effects through hypoxia via Nrf2-ARE pathway have also been found in cardiomyocyte [11,12] and endothelial progenitor cell (EPCs) [13] culture studies. However, these studies focused on effects of hypoxic preconditioning on neuroprotection, cardioprotection, and vascular repair by activation of Nrf2 and expression of its target genes, it remains unknown what happens to the activation of Nrf2-ARE pathway in skeletal muscle after hypoxia exposure.

Hypoxia-inducible factor-1α (HIF-1α) is another key transcription factor which protects cells against hypoxic stress [14]. Researchers have been curious about the role of HIF-1α in regulating Nrf2-ARE signaling pathway, but previous results were not conclusive. Erythropoietin (EPO), one of the target genes of HIF-1α [15], has shown protective effects on rat traumatic spinal cord injury [16] and cultured human SHSY5Y neural cells by activating the Nrf2-ARE signaling pathway [17], whereas another study of Hep3B, HeLa and HEK293 cells has reported that the decreased Nrf2 protein under hypoxia was not recovered by the knockdown of HIF-1 [18], suggesting that HIF-1α did not modulate Nrf2 expression directly. These reported data were mainly collected from *in vitro* pathological tumor cells. There is a lack of direct evidence for HIF-1α affecting Nrf2-ARE signaling pathway in animal or healthy cell models. To the best of our knowledge, there is no study to assess HIF-1α protein expression in the regulation of Nrf2 in animal skeletal muscle and/or C2C12 myoblasts so far, which is closely related to the athletic training.

The purpose of the present study was to investigate the effects of acute hypoxia exposure with different durations on protein expression, transcriptional activity of Nrf2-ARE, and gene expressions of Nrf2-modified antioxidant enzymes, as well as the potential effects of HIF-1α involved in the hypoxia-induced Nrf2 responses. The hypotheses were that the acute hypoxia exposure would increase protein expression, transcriptional activity of Nrf2, and gene expressions of Nrf2-modified antioxidant enzymes, with longer duration associated with higher responses, in which the modulation of HIF-1α would be involved.

## Materials and methods

### Animals and hypoxia exposure

The present study protocols were approved by the Animal Care and Use Committee of Beijing Sport University. Male wild-type (WT) C57BL/6J mice (two months old, n = 36) were provided by the Institute of Laboratory Animal Science of Peking Union Medical College in China. The mice were randomly allocated into four groups: the non-hypoxia control group (0h), 6-hour hypoxia group (6h), 24-hour hypoxia group (24h), and 48-hour hypoxia group (48h), with nine mice in each group. The hypoxia condition was made by placing the mice in a normobaric chamber (210 cm long, 200 cm wide, and 200 cm high). The chamber was infused with hypoxic air through an air compressor and a nitrogen making machine, which could reduce the oxygen concentration in the chamber to 11.2% (at about simulated altitude of 4500 m) based on the previous work [19]. The oxygen concentration in the chamber was monitored with an oxygen sensor. After the hypoxia exposure, the mice were euthanized by cervical dislocation and the quadriceps femoris muscle was collected, cleaned and quick-frozen in liquid nitrogen, and then stored at -80°C.

In order to investigate whether HIF-1α is involved in the hypoxia-induced Nrf2 responses in mouse skeletal muscle, the HIF-1α transgenic (TG) and WT mice were used. Age-matched WT and HIF-1α TG mice were provided by the Institute of Laboratory Animal Science of Peking Union Medical College in China. The TG mice was generated by C57BL/6J mice (background strain of HIF-1α TG mice) and had an overexpression of HIF-1α [20]. The WT and HIF-1α TG mice, with 10 mice in each group (male = 5; female = 5), were two months old with a mean body weight of 21 ± 2 g and were housed with controlled room temperature and lighting (20–25°C and 12:12-h light-dark cycle). They had free access to food and water. After the acclimatization to their housing for one week, the mice were euthanized by cervical dislocation and the quadriceps femoris muscle was collected.

### Culture of C2C12 cells, small interfering RNA (siRNA) transfection and hypoxia treatment

A mouse embryonic myoblastic cell line, C2C12 (ZQ0092), was provided by Zhongqiaoxinzhou Biotech (Shanghai, China). C2C12 cells were grown in Dulbecco’s modified Eagle’s medium (DMEM) (HyClone) supplemented with 10% FBS (Gibco), 100 units/ml penicillin and 100 μg/ml streptomycin (Gibco).

To assess the induced alterations of Nrf2 protein content and the cytosol-to-nuclear translocation of Nrf2, C2C12 cells were also explored in this experimental protocol. For gene silencing, the cells were grown to 70% confluence and then transfected with 22.2 nM (final concentration) of HIF-1α siRNA (siHIF-1α) (sc-35562, Santa Cruz Biotechnology), or non-target specific control scrambled small interfering RNA (siControl) (sc-37007, Santa Cruz Biotechnology) using Lipofectamine RNAiMAX transfection reagent according to the manufacturer’s instructions (Invitrogen). After 48 hours of incubation at normoxia, the transfection medium was replaced with complete medium and then the cells were ready to hypoxic stimulation.

Under the current situation of our laboratory, hypoxia in C2C12 cell culture was designed and achieved by exposing cells to 5% O_2_, 5% CO_2_, and 90% N_2_ in an N_2_-regulated incubator (Thermo 3131, USA) at 37°C. Normoxia (21% O_2_) was used as a negative control during the whole experiments.

### Gene expression using qPCR analysis

Total RNA was isolated from 50 mg of muscle using the TRI reagents (Invitrogen, USA) and C2C12 cell using the TaKaRa MiniBEST Universal RNA Extraction Kit (TaKaRa Bio Inc, Japan) according to the manufacturer’s instructions. Reverse transcription (RT) of total RNA to cDNA was performed using a Reverse Transcription Kit (Toyobo, Japan). RT-PCR was performed in an ABI 7500 Real-Time PCR System (USA) using 10 ul of SYBR Green Real time PCR Master Mix (Toyobo, Japan), 2 ul of cDNA template, 8 ul of mixture of ddH_2_O and respective Qiagen primer sets (Germany) for superoxide dismutase-1 (*Sod1*) (QT00165039), superoxide dismutase-2 (*Sod2*) (QT00161707), Glutamate-cysteine ligase modifier subunit (*Gclm*) (QT00174300), glutamate-cysteine ligase catalytic subunit (*Gclc*) (QT02563995), catalase (*Cat*) (QT010558106), glutathione reductase (*Gsr*) (QT01758232), glutathione peroxydase1 (*Gpx1*) (QT01195936), NAD(P)H quinone dehydrogenase 1 (*Nqo1*) (QT00094367) and *Hmox1* (QT00159915). The mRNA expression levels for all samples were normalized to the level of the house-keeping gene *18S rRNA* (QT010036875). The difference in expression between the control and experimental groups was calculated as 2^-ΔΔCT^, as described previously [21].

### Western blot analysis

Total proteins were isolated from 100 mg of muscle and C2C12 cell using lysis buffer RIPA (P0013B, Beyotime). Protein concentration was measured using the BCA protein assay kit (23225, Pierce, Rockford, IL, USA). The protein expressions of Nrf2 and HIF-1α were detected by Western blot analyses.

After 20 μg total proteins from the muscle samples and 10 μg total proteins from C2C12 cell were separated on a Bolt Bis-Tris plus 4–12% gel (Life Technologies, USA) by electrophoresis, the fractionated proteins were transferred to a nitrocellulose transfer membrane by iBlot Gel Transfer System (Invitrogen, USA). The membrane was blocked for 60 minutes in TBST (Tris-buffered saline with 0.10% Tween 20) containing 5% nonfat milk. The sample was then incubated overnight at 4°C using the following primary antibodies: anti-Nrf2 antibody (sc-722 with skeletal muscle, Santa Cruz Biotechnology; ab62352, with C2C12 cells, Abam company) and anti-HIF-1α antibody (sc-10790, with skeletal muscle; sc-13515 with C2C12 cell, Santa Cruz Biotechnology), and the same blots were stripped and reprobed with β-actin antibody (sc-47778). Density of protein band was analyzed using the Molecular Imager ChemiDoc XRS+ with Image Lab Software (BIO-RAD, USA). The protein levels were normalized to β-actin and then expressed as a fold change of the 0h control group / normoxia group/WT mice group value.

### Analysis of Nrf2 binding with ARE by trans-activation assay

Nrf2/ARE binding efficacy under basal and acute hypoxia conditions were evaluated in the muscle nuclear extracts using a Trans AM Nrf2 transcription factor assay (50296, ActiveMotif, Carlsbad, CA, USA). 10 μg aliquot of total protein was incubated with immobilized oligonucleotides containing the ARE consensus binding site (5’-GTCACAGTACTCAGCAGAATCTG-3’) and the active form of Nrf2 that bound to the oligo was detected using anti-Nrf2 primary antibody after treated with HRP-conjugated secondary antibody. The chromogen formed as a result of specific activity of the transcription factor in the nuclear extracts was determined using a plate reader at 450 nm (BIO-RAD xMark microplate spectrophotometer, USA), and then expressed as a fold change of the control group (0h) or WT mice group value.

### Immunofluorescent staining and confocal microscopy

C2C12 cells were seeded on 35 mm cover glass-bottom culture dishes for 24 hours and were transfected with si-RNA for 48 hours in normoxia, then were treated with hypoxia for 72 hours. The cells were rinsed twice with PBS and were fixed in 4% formaldehyde for 10 min. After washed twice with PBS, the cells were incubated with 0.4% Triton X-100 for their permeabilization and next with PBS/BSA (PBS, pH 7.4, containing 5% BSA) for blocking the non-specific adsorption. Furthermore, following probing with primary mouse anti-Nrf2 antibody (sc-365949, Santa Cruz Biotechnology) overnight at 4°C, the cells were incubated with secondary antibody coupled to Alexa Fluor 555 rabbit anti-mouse IgG (bs-0296, Bioss, Beijing) in 1% BSA for 30 min at room temperature. After cells were washed with PBS again, the nuclei were counterstained with 40-6-diamidino-2-phenylindole (DAPI) (ZLI-9557, ZSGB-Bio, Beijing). Finally, localization of Nrf2 was visualized using a LeicaSP8 laser scanning confocal microscope with a magnification of 400X (Germany) after immunofluorescence staining. Nuclei of the corresponding cells were visualized with DAPI (blue). Images of Nrf2 staining (Orange red) and DAPI staining (blue) of the same area were merged together to locate the cells with nuclear Nrf2 accumulation.

### Statistical analysis

All values were reported as means ± SE. Statistical calculations were performed using SPSS13 (SPSS Inc., Chicago, IL, USA). Data were analyzed by independent-sample Student’s t test or one-way ANOVA. The level of statistical significance was set at *p*<0.05.

## Results

### Effects of acute hypoxia exposure with different durations on HIF-1α and Nrf2 protein contents

**Fig 1:**
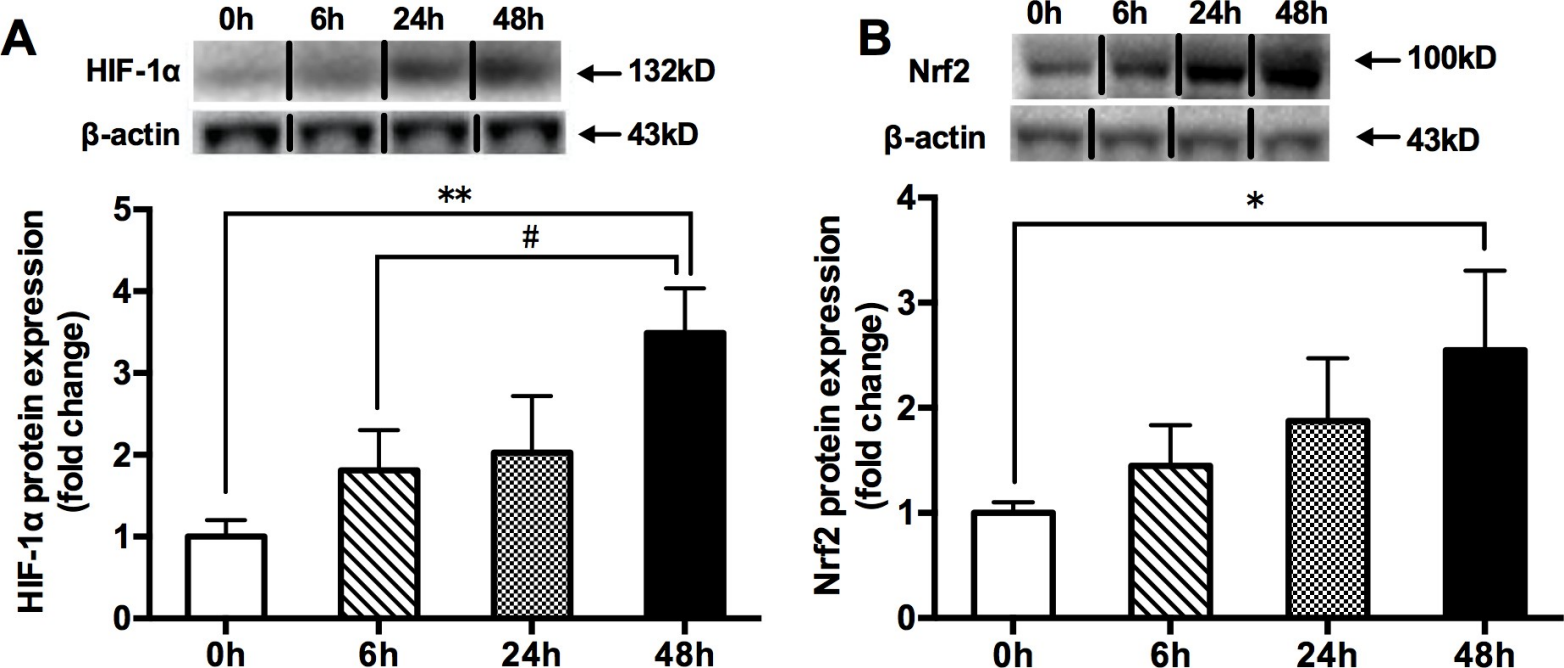
**The changes in HIF-1α (A) and Nrf2 (B) total protein contents in mouse** **quadriceps** **femoris**. Muscles were collected following acute exposure to hypoxia with different durations (n = 9 mice/group). Protein expression was measured by western blotting. The values are expressed as the means ± SEM. **p<0.01 or *p<0.05 vs. 0h group; #p<0.05 vs. 6h group.

Compared with the 0h hypoxia group, the 48h hypoxia significantly increased the protein expressions of HIF-1α and Nrf2, but the 6h and 24h hypoxia did not (Fig 1).

### Effects of acute hypoxia exposure with different durations on Nrf2/ARE binding activity and the mRNA expressions of Nrf2-mediated antioxidative genes

**Fig 2:**
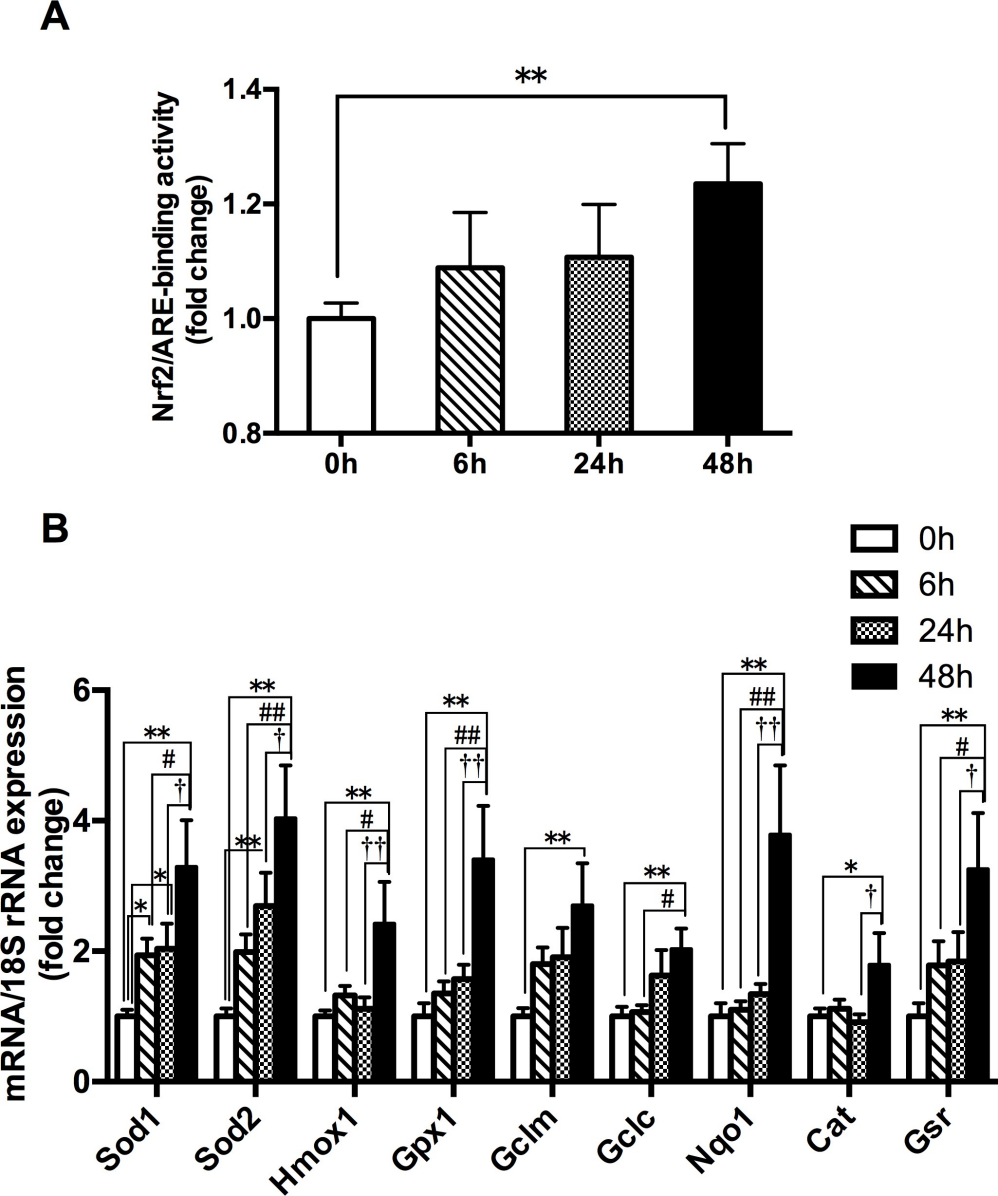
**The changes in the Nrf2-ARE binding activity (A) and the mRNA expressions of Nrf2-mediated antioxidative gene (B) in mouse** **quadriceps** **femoris**. Muscles were collected following acute exposure to hypoxia with different durations (n = 9 mice/group). Nrf2-ARE binding activity was measured by trans-activation assay. The mRNA expressions were measured by RT-PCR. The values are expressed as the means ± SEM. **p<0.01 or *p<0.05 vs. 0h group; ##p<0.01 or #p<0.05 vs. 6h group; ††p<0.01 or †p<0.05 vs. 24h group.

The 48h hypoxia group produced a significant increase in the Nrf2-ARE binding activity and the mRNA expressions of Nrf2-mediated antioxidative genes (*Gclm*, *Gclc*, *Sod1*, *Sod2*, *Cat*, *Gpx1*, *Nqo1*, *Gsr* and *Hmoxo1*) in skeletal muscle, while no changes in the Nrf2-ARE binding activity and most of the mRNA expressions of Nrf2-mediated antioxidative genes were found in the 6h and 24h hypoxia groups, compared with that of the 0h control group (Fig 2). Taken together, with longer hypoxia duration associated with higher Nrf2 responses, the 48h hypoxic exposure could activate the Nrf2-ARE pathway (Figs 1 and 2).

### Alterations of HIF-1α protein expression after acute hypoxia exposure and inhibition of the HIF-1α impaired hypoxia exposure (HE)-mediated responses through Nrf2 activation in C2C12 cells

**Fig 3:**
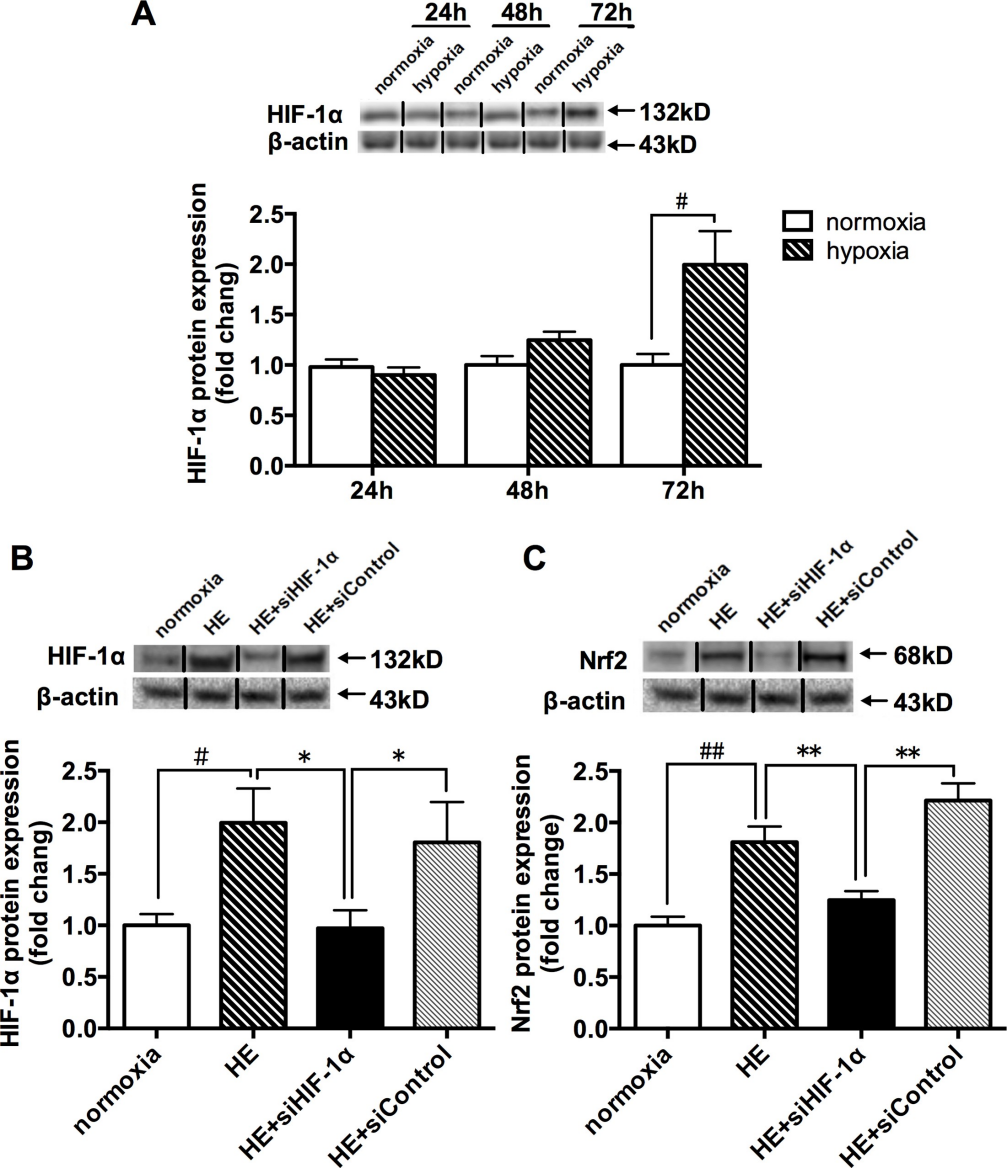
**Alterations of the protein expressions of HIF-1α at different time points after hypoxia exposure (A); siRNA-mediated knockdown of HIF-1α inhibited HIF-1α (B) and Nrf2 (C) protein contents in C2C12 cells.** Anti-HIF-1αsiRNA (si-HIF-1α,) and its control siRNAs (siControl) were transiently transfected into C2C12 cells before HE. Cells were incubated in normoxia (21% O_2_) or hypoxia (5% O_2_) and protein content levels of relevant molecules were measured by western blotting. The values are expressed as the means ± SEM. **p<0.01 or *p<0.05 vs. HE or HE+siControl group; ##p<0.01 or #p<0.05 vs. normoxia group. HE = 72h hypoxia exposure at 5% O_2_.

**Fig 4:**
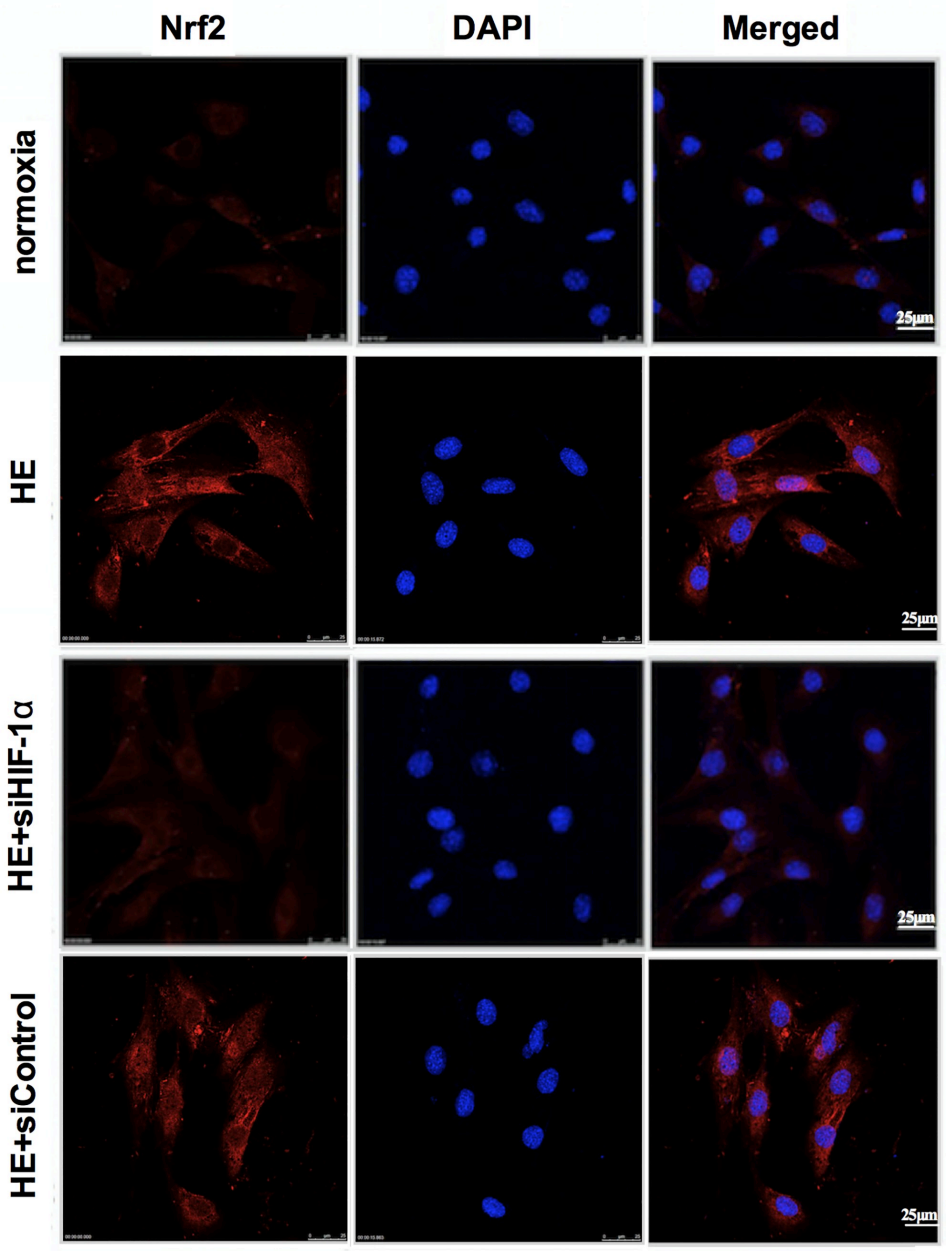
Effects of HE and HE+si HIF-1α on the nuclear distribution of Nrf2 in C2C12 cells. Cells were incubated in normoxia (21% O_2_) or HE (5% O_2_) and then localization of Nrf2 was visualized with a fluorescence microscope after immunofluorescence staining with anti-Nrf2 antibody and rabbit anti-mouse IgG second antibody coupled to Alexa Fluor 555. The nuclei were counterstained with DAPI (blue). Images of Nrf2 staining (Orange red) and DAPI staining (blue) of the same area were merged together to locate the cells with nuclear Nrf2 accumulation. HE = 72h hypoxia exposure at 5% O_2_.

In order to identify the effects of hypoxia exposure (5% O_2_) with different durations on HIF-1α protein contents in C2C12 cells, the protein expression was measured after hypoxia exposure for 24h, 48h and 72h, respectively. We found that at least 72 hours were needed to obtain the significant HIF-1α protein expression (Fig 3A). Therefore, in the following cell experiments, we used 72 hours of hypoxia as the basic treatment. The ‘HE’ in Fig 3B and 3C, and Fig 4 represented 72 hours of hypoxia exposure. There were four groups in the cellular studies: normoxia, HE, HE+siControl, and HE+siHIF-1α. After the 72h hypoxia treatment, the cells were harvested. Protein expression levels of relevant molecules were analyzed by western blotting and Nrf2 nuclear translocation was detected by immunofluorescence staining and confocal microscopy.

The results shown that the protein levels of HIF-1α and Nrf2 were markedly increased and the Nrf2 located primarily in a cytosolic location was translocated to the nucleus in the C2C12 cells in the HE group in contrast with the normoxia group, implying that the HIF-1α and Nrf2 was activated after the 72h hypoxia treatment (Fig 3B and 3C, Fig 4). Moreover, the molecule of HIF-1α was blocked to validate its regulatory role in HE-mediated effects on Nrf2. Anti-HIF-1α siRNA (si-HIF-1α) and its control siRNAs (siControl) were transiently transfected into C2C12 cells before the 72h hypoxia treatment. The data also revealed that the HE+si-HIF-1α group exhibited a significant reduction in HIF-1α and Nrf2 protein contents, and that the cytosol-to-nuclear translocation of Nrf2 was prevented, compared with the HE and HE+siControl groups (Fig 3B and 3C, Fig 4). Collectively, the aforementioned results supported that HE-mediated effects on Nrf2 could be partially ascribed to activation of the HIF-1α.

### Protein expressions of HIF-1α and Nrf2, Nrf2/ARE-binding activity and the mRNA expressions of Nrf2-mediated antioxidative genes in skeletal muscle of WT and HIF-1α TG mice

**Fig 5:**
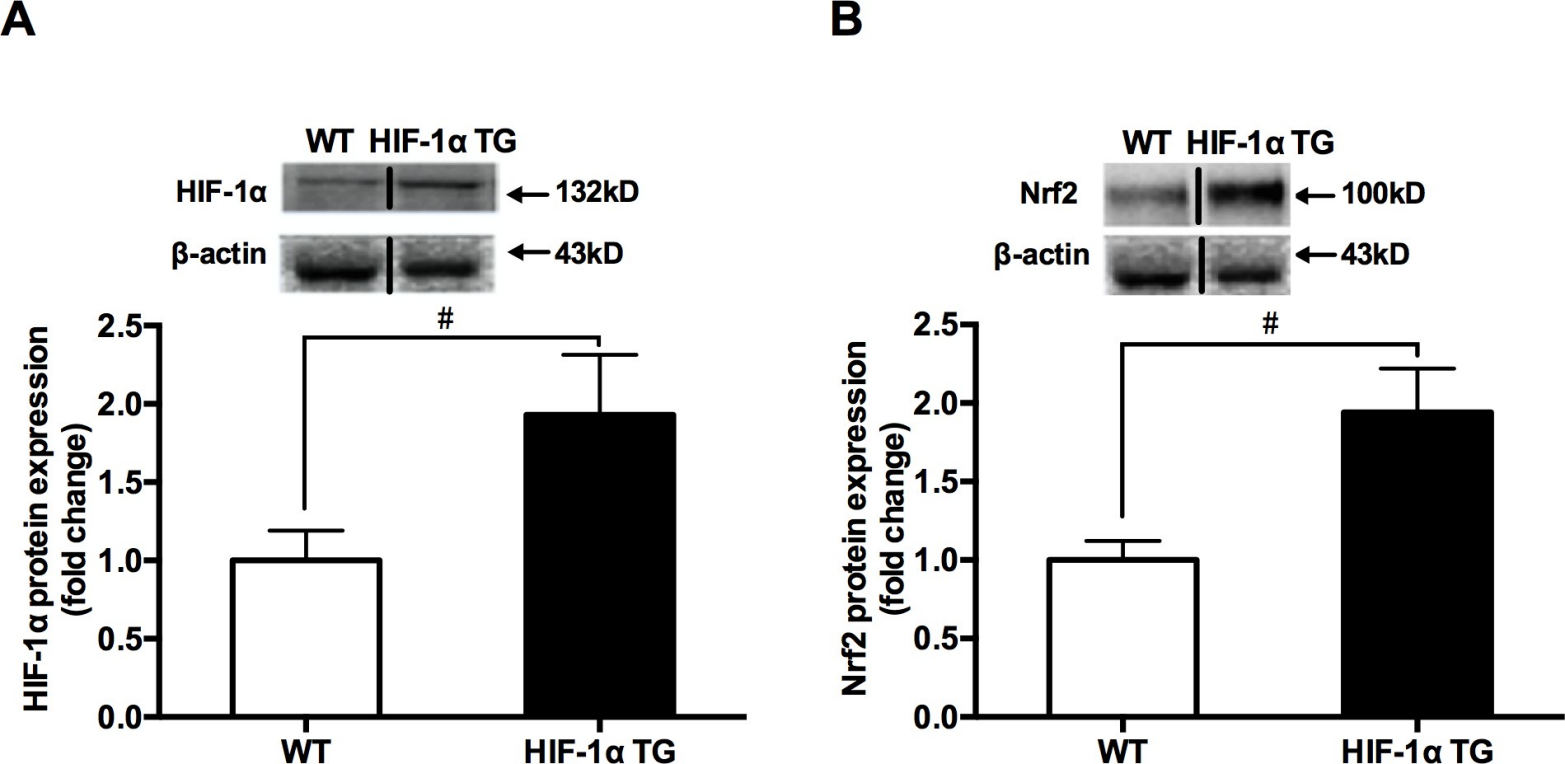
**The changes in HIF-1α (A) and Nrf2 (B) total protein contents between HIF-1α TG and WT mice groups.** Mouse quadriceps femoris muscles were collected (n = 9 mice/group). Protein expression was measured by western blotting. The values are expressed as the means ± SEM. #p<0.05 vs. WT mice group.

**Fig 6:**
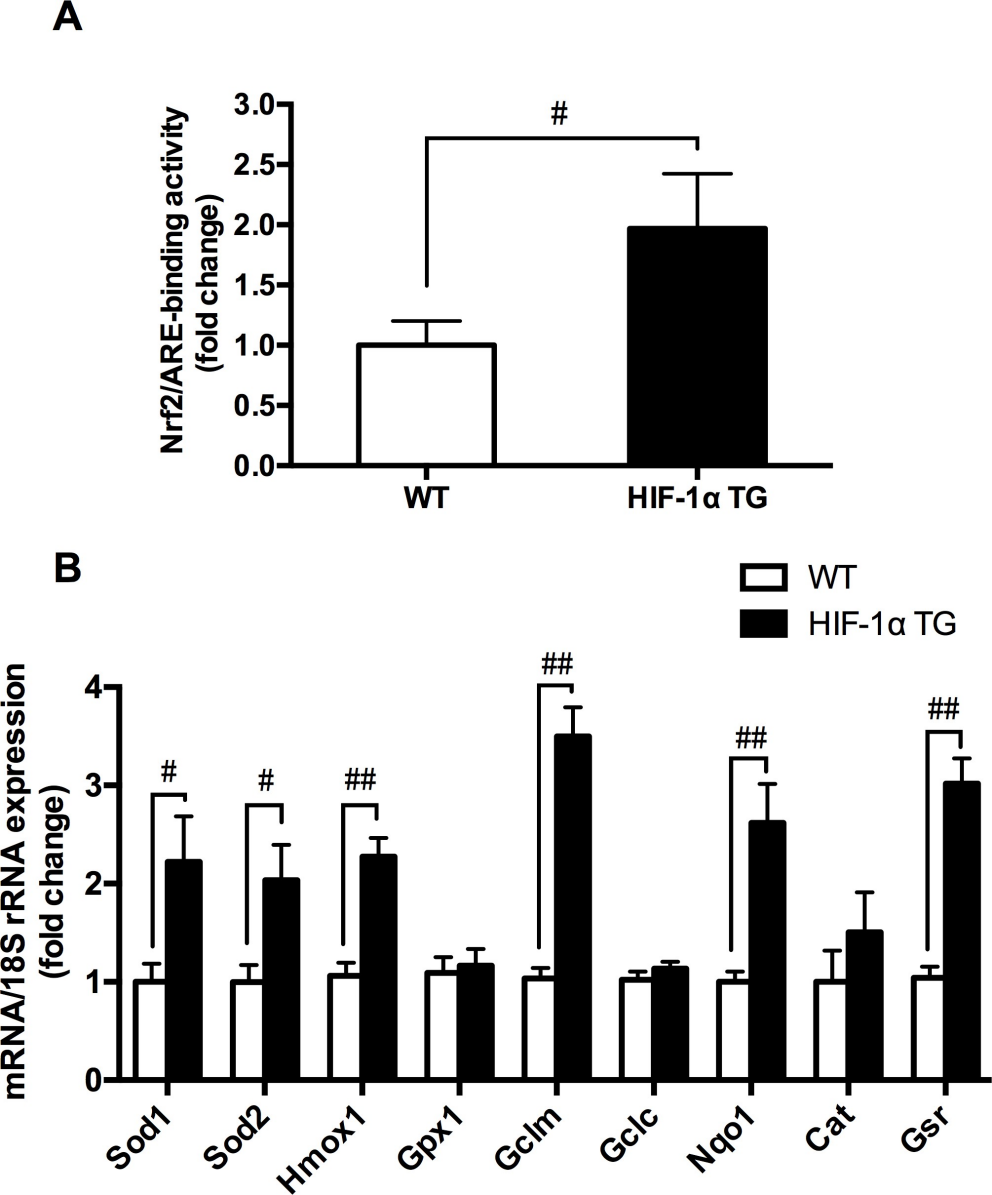
**The changes in Nrf2-ARE binding activity (A) and the mRNA expressions of Nrf2-mediated antioxidative genes (B) between HIF-1α TG and WT mice groups.** Mouse quadriceps femoris muscle was collected (n = 9 mice/group). Nrf2-ARE binding activity was evaluated in the muscle nuclear extracts using a Trans AM Nrf2 transcription factor assay. The level of mRNA expression was measured by RT-PCR. The values are expressed as the means ± SEM. #p<0.05 or ##p<0.01 vs. WT mice group.

To determine the effects of HIF-1α on the Nrf2-mediated antioxidant responses in mouse skeletal muscle, the HIF-1α TG and WT mice were also used. The protein content levels of HIF-1α and Nrf2 measured by western blotting were compared in between HIF-1α TG and WT mice. The results showed that the levels of HIF-1α and Nrf2 protein contents (Fig 5), Nrf2/ARE-binding activity, and the mRNA expressions of Nrf2-mediated antioxidative genes (Fig 6) in skeletal muscle of HIF-1α TG mice were significantly higher, compared with those in WT mice, suggesting that HIF-1α could be involved in hypoxia exposure-induced Nrf2-mediated antioxidant responses.

## Discussion

The main findings of the present study were that the 48h hypoxia significantly increased Nrf2-ARE binding activity, Nrf2 protein, and mRNA expressions of Nrf2-mediated antioxidant genes in mouse skeletal muscle, longer duration associated with higher responses, in which the modulation of HIF-1α would be involved; while the 6h and 24h hypoxia did not achieve these changes. The overall results supported the hypotheses of the present study. As far as we know, this is the first study to examine the changes in activation of Nrf2-ARE pathway in skeletal muscle after acute hypoxic exposure with different durations.

Hypoxia can stimulate Nrf2 signaling in animal or human tissues [10] or cells [11–13,22]; however, the activation process is complex. Several stress-mediated cytosolic kinases: phosphoinositide 3-kinase (PI3K), protein kinase C (PKC), and mitogen-activated protein kinase (MAPK), have been reported to modify Nrf2 and affect its transcription activity [23–25]. For example, hypoxia activated PI3K/Akt pathway to mediate hypoxia-induced Nrf2 activation in EPCs [13] and enhanced the phosphorylation of serine residues of Nrf2 by PKC [18]. In addition, dissociation of Nrf2 from Kelch-like ECH-associated protein 1 (Keap1) is the first step for nuclear translocation and subsequent DNA binding of Nrf2 to regulate the inducible expression of cytoprotective genes [26]. Before the activation, Nrf2 is seized in the cytosol as an inactive complex with its repressor Keap1 [27]. Thus, these evidences imply that the activation of Nrf2-ARE pathway may need a certain time. In the present study, hypoxia exposure for 6h and 24h did not change Nrf2 protein expression, Nrf2/ARE binding activation and Nrf2’s downstream target gene mRNA expression; while hypoxia exposure for 48h promoted the relevant changes in skeletal muscle of WT mice (Fig 1B and Fig 2). These results suggested that acute hypoxia exposure as a non-pharmacological approach, like exercise [28], could also activate Nrf2/ARE signaling pathway in skeletal muscle. Under the current experimental condition, 48 hours of hypoxic stress might be required for the break of Nrf2 from Keap1 and then start the Nrf2-dependent antioxidant response.

Higher expression of HIF-1α in mouse skeletal muscle in response to hypoxic stimuli is consistent with the previous reports [29,30]. Interestingly, the highest level of HIF-1α expression was found in the 48h group, with same pattern of change as Nrf2 protein expression and the mRNA levels of antioxidative genes involved in Nrf2/ARE binding activity. Therefore, these evidences seemed to indicate that HIF-1α might involve in the hypoxia-mediated regulation of Nrf2 activation. In order to get a deeper understanding of the effects of HIF-1α on the hypoxia-induced Nrf2 responses in skeletal muscle, we employed C2C12 myoblasts with HIF-1α-specific siRNA to determine whether HIF-1α plays an important role in Nrf2 signaling pathway under hypoxia. It was shown that HIF-1α knockdown cells displayed a remarkable inhibition of Nrf2 protein along with a concomitant translocation of Nrf2 into the nuclei in HE. We noticed a methodological issue about hypoxic condition in C2C12 cell culture, other research teams used 1% O_2_ to treat the cells [31–33]; however, with the current equipment in our laboratory, we could only conduct the hypoxia exposure at 5% O_2_. The results, nevertheless, have achieved the experimental design of the present study. In the present study, C2C12 cells treated at 5% O_2_ needed to take 72 hours to significantly increase the protein expression of HIF-1α. Furthermore, the different changes in Nrf2 protein contents, Nrf2/ARE-binding activity and the mRNA expressions of Nrf2-mediated antioxidative genes were compared between HIF-1α TG and WT mice. The data indicated that with the increase of skeletal muscle HIF-1α protein expression in the HIF-1α TG mice, their Nrf2 protein content, Nrf2/ARE-binding activity and the mRNA expressions of Nrf2-mediated antioxidative genes were also upregulated. Taken together, these evidences support that HIF-1α may play an important part in the Nrf2-mediated antioxidant responses in mouse skeletal muscle.

We should admit the limitations in this study. We only focused on the effects of acute hypoxia exposure on the activation of Nrf2/ARE antioxidant signaling and whether HIF-1α expression involved in promoting the Nrf2-mediated antioxidant responses in mouse skeletal muscle, but did not measure the change levels of reactive oxygen species (ROS) and oxidative stress products, such as malondialdehyde (MDA). It is worth to mention, besides Nrf2, nuclear factor-κB (NF-κB) is also a key transcription factor that may regulate cellular responses to oxidative stress and inflammation, and there is functional cross-talk between these two important pathways [30]. Future researches are required to elucidate the changes of redox homeostasis in the molecular mechanisms that link the Nrf2 and NF-κB pathways after hypoxia exposure by using Nrf2 knockout mice.

In conclusion, our data demonstrated with molecular evidence that Nrf2/ARE antioxidant signaling activation in mouse skeletal muscle is dependent on hypoxia duration. Exposure to hypoxia for 48h triggers the activation of Nrf2 and is a positive regulator. HIF-1α protein expression may be involved in promoting the Nrf2-mediated antioxidant responses, which partly underlies the beneficial effect of hypoxia in promoting Nrf2 and upregulating ARE-dependent antioxidant transcription.

## Supporting information

S1 Data Data file. (XLSX)
